# Dietary consumption of beef and red meat: a scoping review and evidence map on cognitive outcomes across the lifespan

**DOI:** 10.1017/S1368980023001933

**Published:** 2023-12

**Authors:** Tristen L Paul, Stephen A Fleming

**Affiliations:** Traverse Science, 435 E Hawley Street #816, Mundelein, IL 60060, USA

**Keywords:** Beef, Red meat, Dietary intake, Cognition, Scoping review

## Abstract

**Objective:**

Mixed evidence exists on the impact of beef consumption on cognition. The goal was to create an evidence map capturing studies assessing beef consumption and cognition to reveal gaps and opportunities in the body of literature.

**Design:**

A scoping review was conducted to locate studies up to March 2022 using PubMed and backwards citation screening. Data were extracted by two independent reviewers with conflict resolution, and a database was created and made publicly available.

**Setting:**

Intervention and observational studies.

**Participants:**

Humans of any age, sex and/or health status, without moderate to severe cognitive impairment and/or abnormalities.

**Results:**

Twenty-two studies were identified that quantified beef or red meat intake and assessed cognition. Six studies assessed beef intake, with the remaining studies describing intake of red meat that may or may not include beef. Nine articles described randomised controlled trials (RCT), mostly conducted in children. Thirteen described observational studies, primarily conducted on adults and seniors. The most common cognitive domains measured included intelligence and general cognition, and memory. The majority of controlled studies were rated with high risk of bias, with the majority of observational trials rated with serious or greater risk of bias.

**Conclusions:**

Red meat and beef intake and cognition is largely understudied. There is a significant lack of replication across study designs, populations, exposures and outcomes measured. The quality of the research would be considerably enhanced by focused assessments of beef intake (and not red meat in general) and specific cognitive domains, along with improved adherence to reporting standards.

Cognitive development, defined as the acquisition, organisation and learned use of knowledge, occurs in stages spanning infancy through adulthood^(1)^. While infancy and early childhood are considered critical stages for brain development^(2)^, nutrition may exhibit protective effects against the onset and development of cognitive impairment throughout the ageing process^(3–5)^. The importance of adequate nutrition and nutrient status across the lifespan is emphasised through the extensive research on macro- and micronutrient intake^(6–9)^ and is reflected in the ill effects of malnutrition on general development^(10)^. Iron, for example, is an essential mineral involved in central nervous system development, and a deficiency at any life stage could prove detrimental to neurophysiological function^(11)^. Another key nutrient in the developmental process, choline, has been shown to prevent neural tube abnormalities during fetal development and improve neurocognitive development into early childhood when consumed at higher quantities during pregnancy^(12–17)^. Animal-source foods provide a high quantity and bioavailability of these essential nutrients^(18,19)^ and are particularly rich in *n*-3 fatty acids, along with vitamin A and D, zinc, and iodine^(20)^. Animal-source foods further provide adequate proportions of each of the nine essential amino acids required in the human diet, making them a complete protein^(20)^. Beef is one such food containing high-quality, readily available, and absorbable micro- and macronutrients^(21)^. Many of the micronutrients in beef are considered a requirement for normal growth, activity, and health and are further known to impact cognitive development^(2)^. Moreover, dietary patterns containing beef have been shown to contain significant amounts of vitamins and minerals in the diet, including vitamin B_6_ and B_12_, zinc, choline, niacin, phosphorus, potassium, and magnesium^(22)^.

Despite the wealth of research providing evidence for the beneficial effects of the components in beef for brain development, there is conflicting evidence on the impact of beef intake itself on cognition. Two observational studies, one with elderly subjects with no risk of cognitive impairment^(23)^ and another on those with at least mild cognitive impairment (MCI)^(24)^, found that dietary patterns higher in red meat intake were associated with increased risk of cognitive impairment. Similarly, Yuan *et al*.^(25)^ reported that patients with MCI consumed more red meat daily. Alternatively, Hepsomali and Groeger^(26)^ found that higher unprocessed red meat intake (but not processed meat) was associated with improved general cognitive ability, and Zhu *et al*.^(27)^ reported that red meat intake (40–60 g/d) was associated with reduced probability of mental impairments later in life. While the above-mentioned studies report conflicting results, two studies^(28,29)^ presented neutral results, finding no association between red meat intake and MCI. Their findings are in agreement with Hawley and colleagues, who recently performed a review of nine RCT performed in older and elderly adults and found no impact of beef consumption or the consumption of nutrients found in beef on cognition^(30)^. However, and to the point of this manuscript, only one study^(31)^ of the nine identified in Hawley’s review evaluated beef as a whole food, but this study did not measure cognition. A recent review by An *et al*.^(32)^ further provided evidence on the heterogeneity of results when considering beef intake and cognition in children.

As a means of assessing the current literature describing beef and red meat consumption in humans and cognition-related outcomes, an evidence map was created to characterise the degree to which studies quantify and report beef and/or red meat consumption and the forms in which they are consumed. The primary objective of this effort was to identify sources of heterogeneity, assess risk of bias and find available data across a range of beef intakes to capture gaps in the literature that may inform future research efforts. Results and possible dose–response relationships between these and the exposure of interest were not explored.

## Methods

This scoping review was registered in PROSPERO with ID: CRD42022350093 and conducted in accordance with the Preferred Reporting Items for Scoping Review (PRISMA) recommendations^(33)^. Amendments to the original protocol are available and can be viewed on the PROSPERO registry.

### Study selection and inclusion

Studies that met the following criteria were included in the review: studies that (1) were performed on humans of any age, sex, and/or health status (except those with brain injury, neurodegenerative diseases, or moderate-to-severe cognitive impairments); (2) reported the consumption of quantified amounts of beef and/or red meat where the ‘red meat’ category could reasonably be assumed to contain beef; (3) quantified red meat or beef intake such that a g/d amount was calculable (excluding instances where categorical ranges were provided, such as consuming <1 serving of beef/week, or eating beef >5 times/week); (4) measured and reported outcomes related to cognition in relation to beef and/or red meat consumption; (5) published primary research in the form of controlled trials or observational studies; (6) were published in English; and (7) had available full texts. Studies were excluded if: (1) they were *in vitro* or non-human studies; (2) the subjects had significant cognitive deficits at baseline other than MCI; (3) meat intake was not defined or quantified; (4) they evaluated isolated components of beef and/or red meat (e.g. Fe, carnosine and fatty acids); (5) beef/red meat intake was reported as a composite variable of dietary intake (e.g. a principal component analysis was performed on dietary intake, but intake was reported as a factor loading) or intake was measured but not reported (e.g. the study reported regression coefficients of beef intake against cognition but did not report intake on a basis that could be converted to g/d); and (6) outcomes were not directly related to cognition (examples include but are not limited to: appetite, depression, sleep, mood, brain injuries (e.g. stroke and traumatic brain injury), or neurodegenerative diseases such as Alzheimer’s or Parkinson’s disease).

### Search strategy

The search strategy employed the use of both a search engine and extensive backwards citation screening. First, a systematic literature search was performed on the 28 March 2022 in the National Library of Medicine’s PubMed database with no date restriction and filtered to include only literature in humans. Medical subject headings (MeSH) and text terms were entered to ensure that all relevant indexed and non-indexed literature was captured in the search. Search terms are provided in Supplemental Table 1. Studies were independently screened by two reviewers blinded to each other using Rayyan.ai (https://www.rayyan.ai/). All results from the search were screened at the title/abstract level, and upon meeting inclusion criteria at this stage were further screened at the full-text level. A list of the studies excluded at full-text screening is available in Supplemental Table 2.

Upon completion of screening literature from PubMed, backwards citation screening was used to identify additional literature. Given relatively few articles were returned from the PubMed search, we chose to use backwards citation screening instead of searching through additional databases. A backwards citation search was performed on each in-scope article, whereby reference lists were scraped in their entirety for relevant literature. If a new study was included from a reference list, the reference list of that study was also scraped for additional relevant articles until no additional relevant articles from the citation lists of previously included articles were present. Additionally, references from select reviews were scraped for in-scope articles^(5,32,34–50)^. The relevance of articles for inclusion from reference lists was primarily based on the title, whereby if considered potentially in-scope, the abstract and full text were consulted to confirm inclusion. Determination of relevant reviews to be scraped was based on inclusion of the exposure and outcomes of interest evaluated in that review. Results from backwards citation screening were single-reviewed and in the case of uncertainty, discussed between reviewers until a consensus was reached.

### Data extraction

Data were collected independently by two reviewers in Microsoft Excel using custom data extraction procedures and forms. Predefined variables were collected either at the study- (article, subject, study design and outcome information) or group-level (exposure type and intake information). Direction of effect for outcome measures were not extracted. A full list and description of each variable included in the database is available in Supplemental Table 3. Any conflicts which arose at screening or data extraction were resolved by discussion between reviewers after all data were extracted, or by the opinion of a third reviewer in the instance of a persistent disagreement among reviewers.

Based on how authors defined red meat intake, measurements were binned into one of three categories: ‘beef only’, whereby authors reported subjects to have consumed red meat as beef and no other red meats; ‘red meat includes beef’, whereby beef formed part of red meat intake, however other red meats such as mutton and pork were also specified; and ‘red meat unspecified’, whereby no indication of the source of red meat was specified.

In order to increase comparability between studies, the intake of beef or red meat was collected as reported in the study, and if that was not in units of g/d (e.g. reported servings/week), units were translated to g/d as follows: any measurement reported per week or month was divided by seven or thirty to convert to a per-day basis. To convert servings to grams, servings were converted to oz by multiplying by 3 based on the reference amount customarily consumed (RACC) of 3 oz/serving^(51)^; and then oz were converted to grams by multiplying by a conversion factor of 28·34. Where studies reported consumption as meals per week^(52,53)^, one meal was assumed to be equivalent to one serving. Where studies reported consumption as times per week^(29)^ it was assumed that each meal or consumption time equated to one serving. One study reported beef intake as baked into biscuits, that is, biscuits/week^(54)^. Beef intake in g/d was back-calculated from the provided amount of protein in the biscuit and subsequent matching to that of the protein content of a similarly described beef strip item (code 13350) from the US Department of Agriculture FoodData Central database. An additional study^(55)^ reported consumption as both a range (less or greater than 50 g/d) and discrete averages, and only the averages were extracted.

Due to the heterogenous nature in the reporting of outcome measures, outcomes were binned into one or more of the following nine cognitive domains: memory, psychomotor, language and verbal function, auditory and visual function, executive function, spatial reasoning, intelligence and general cognition, attention, and numeric cognition. The tool used was then categorised according to whether it was designed to assess general cognitive function or impairment (global tool), or to specifically measure a given psychological construct or task performance (domain-specific tool). For example, the Mini-Mental State Examination (MMSE) was considered a ‘global’ tool, whereas the Ravens Progressive Matrices task was considered domain-specific. See online Supplemental Table 4 to see which tools were assigned a global or domain-specific grouping.

### Risk of bias assessment

Risk of bias assessment was performed by two independent reviewers. Intervention trials were assessed with the RoB 2.0 tool^(56)^. Observational trials were assessed using the RoBNObs tool, an unpublished tool developed and used in the 2020 Dietary Guidelines for Americans^(57)^. For RCT, the overall risk of bias was determined as follows: trials received an overall rating of ‘low risk’ if the trial was rated as ‘low’ across all five domains, an overall rating of ‘some concerns’ if at least one of the five domains were rated as ‘some concerns’, and an overall rating of ‘high risk’ if at least one of the domains were rated as ‘high risk’ or where four or more individual domains were rated as ‘some concerns’. Observational trials were assessed and assigned an overall risk rating based on the same concept. For example, studies were rated as overall ‘serious risk’ if at least one of the seven individual domains were rated as ‘serious’ or where four or more individual domains were rated as ‘moderate’. Any conflicts which arose were resolved by discussion between the reviewers or final assessment of the senior reviewer.

### Visualisation and database

Risk of bias figures were visualised in Microsoft Excel. Beef and red meat intake concentrations and cognitive outcome figures were visualised using RStudio version 3.6.2 (http://www.rstudio.com/). This database has been made publicly available with links to an interactive visualisation at https://github.com/Traverse-Science/Beef-and-Cognition-SR.

## Results and discussion

**Fig. 1:**
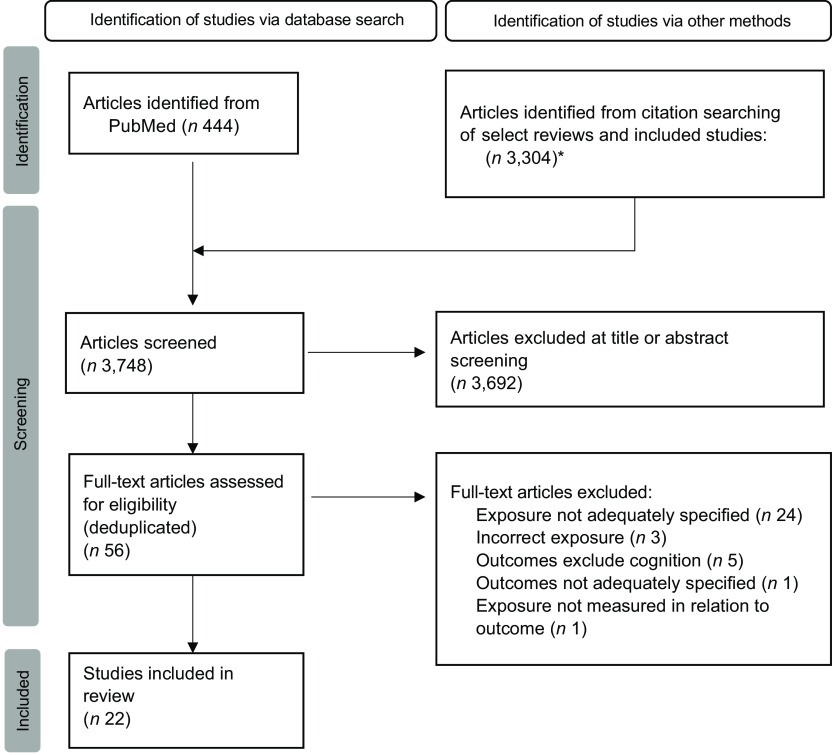
PRISMA diagram. ^*^Backwards citation screening was conducted by a single reviewer (at the title and abstract level). All other steps were conducted through dual review and conflict resolution

A total of 3,748 articles were screened for inclusion and twenty-two were included (Fig. 1), where ten articles were captured in the database searches and an additional twelve were found through backwards citation searching. Of the twenty-two studies, nine were intervention trials and thirteen were observational studies.

### Terminology

The heterogeneity of terms used to report beef and red meat is displayed in Table 1. ‘Beef only’ intake was reported exclusively in RCT, with no observational studies reporting this intake category. Further, four of the RCT reported from the same parent study, The Child Nutrition Kenya Project (CNP), a 2-year, cluster-randomised trial during which Kenyan school children were fed a traditional stew with either added ground beef, vegetable oil or with a glass of milk. Thus, for the ‘beef only’ category, ground beef was the most common form reported here. Despite minced beef and finely ground beef being similar, the current layout highlights the diversity in reporting, particularly within the forms of beef. In addition to describing the form of beef, reporting the fat content and cooking method varied but was usually omitted. Three authors described beef with terms such as ‘lean’, ‘10–12 % fat’ or ‘roast’^(28,58,59)^. The discrepancy in how beef and red meat food groups are reported, as well as the dissimilarity between studies regarding specific descriptions of the type and quality, impedes the ability to draw accurate conclusions on intake and cognitive outcomes. Improved standardisation of terminology and descriptions is required to allow for such comparisons to be made, which in turn will improve researcher’s ability to associate intake data with chronic disease and cognition-related outcomes.

**Table 1:** Red meat categories and forms as reported in included studies

Category	Studies *n*	%	Forms	*n*
Beef only	6	27	Ground beef	2
			Dried beef powder from blended dried beef strips	1
			Finely ground beef (with 10–12 % fat)	1
			Beef (eye round roast, top sirloin, roast beef sliced, ground beef 90 % lean, pot roast and beef short loin)	1
			Minced beef	1
Red meat, includes beef	8	36	Beef, pork and mutton	2
			Beef, pork and lamb	1
			Lean red meat (varied cuts of beef, lamb and veal trimmed of visible fat)	1
			Beef or mutton	1
			Red and processed meats (beef, pork, or lamb as a main dish or in mixed dishes, hamburgers, hot dogs, bacon and other)	1
			Meat (chicken, lamb and beef)	2
Red meat, unspecified	8	36	Red meat	6
			Red and processed meats	1
			Red meat and meat products	1
			Red meat, fresh*	1
			Red meat, organ*	1
			Red meat, preserved*	1

* Separate categories are reported from the same study.

Where beef was specified as part of the red meat category, terminology describing ‘beef, pork, and mutton/lamb’ dominated amongst the included studies. Organs and preserved variations of red meat were amongst the least reported forms within the ‘red meat, unspecified’ category (reported once each); however, these forms were reported from the same study^(23)^, together with a ‘red meat’ category without further specification. This evidence further revealed the lack of information and inadequate reporting surrounding red meat quality. For example, the majority of studies were within the ‘red meat, unspecified’ category and seldom reported parameters describing the nutrient quality of red meat.

During screening and full-text review, it was largely unclear what animal protein sources were included in calculations of meat or red meat intake, and if meat included sources such as poultry or fish. For example, one study (included as two separate articles)^(52,53)^ included ‘chicken’, together with lamb and beef, as part of the meat-receiving group’s treatment diet, but also specified how much red meat was consumed during dinners. This further affirms the difficulty in translating findings between studies. A review by O’Connor^(60)^ revealed that although the number of publications reporting on beef or red meat has increased over time, the reporting of details has shown little improvement. Screening of studies also revealed that although beef and red meat is often ‘quantified’, that is, assigned a value, the reported values are not necessarily intake at a calculable level and therefore cannot be utilised for comparisons across studies. For example, one study which was excluded at the full-text level assigned beef a factor loading as part of either a processed or Mediterranean dietary pattern^(61)^. Another study reported intake for red meat as a rotated component matrix^(62)^. Although this data initially appeared relevant to the evidence base relating beef intake and cognition, the reporting of intake in this manner does not allow the actual amount consumed to be known or derived/calculated, as to allow the translation of findings within and across studies. Quantification of intakes that are not translatable to the actual amount consumed creates a false impression with regard to the size of the body of evidence associating beef intake and cognition, in that it is much larger than what it truly is. The reporting of intake in a format that adequately describes the exposure and is calculable would largely increase the amount of available data and allow increasingly accurate guidance to be made.

### Study characteristics

#### Intervention trials

Of the twenty-two included studies, nine were intervention trials, four of which are reports from the CNP^(59,63–65)^ (Table 2). Three additional trials evaluated children, one of these similarly being located in Kenya^(54)^ and two reports from the same parent study conducted in Norwegian preschool children^(52,53)^. The remaining interventions evaluated adults^(58)^ and seniors^(28)^. The only intervention study to restrict sex to females was performed in the USA^(58)^. At baseline, none of the intervention trials selected for or included subjects with cognitive-related impairments or risks. Sample sizes at randomisation ranged from 43 to 900, with the majority of the intervention studies providing the treatment for approximately 2 years (from the CNP), closely followed by between 3 to 4 months. Excluding the CNP, there was almost an equal number of independent trials who had intervention durations of less than 1 year. Intervention trials studied subjects from a wide scope of geographical locations; however, Asian and South American populations were not represented.

**Table 2:** Study characteristics of intervention trials

Reference	Characteristic			Red meat category			
		Total count		Beef only		Red meat, includes beef	
		*n*	%*	*n*	%*	*n*	%*
		9	100	6	67	3	33
Design							
^(28,52–54,59,63–65)^	RCT, parallel arm	9	100	6	100	3	100
Age category							
^(52–54,59,63–65)^	Child (3–11 years)	7	78	5	83	2	67
^(58)^	Adult (18–64 years)	1	11	1	17	0	
^(28)^	Senior (65+ years)	1	11	0		1	33
Cognitive status at baseline							
	MCI or risk of CI	0		0		0	
^(28,52–54,58,59,63–65)^	No impairments	9	100	6	100	3	100
Participant region							
^(54,59,63–65)^	Africa	5	56	5	83	0	
^(28)^	Australia	1	11	0		1	33
^(52,53)^	Europe	2	22	0		2	67
^(58)^	North America	1	11	1	17	0	
Sample size							
^(28,52–54,58)^	0–249	5	56	2	33	3	100
^(64)^	250–499	1	11	1	17	0	
^(63,65)^	500–749	2	22	2	33	0	
^(59)^	750–1000	1	11	1	17	0	
Duration							
^(52,53,58)^	>3–4 months	3	33	1	17	2	67
^(28)^	>4–6 months	1	11	0		1	33
^(54,63–65)^	<1–2 years	4	44	4	67	0	
^(59)^	>2 years	1	11	1	17	0	

RCT, randomised controlled trial; MCI, mild cognitive impairment; CI, cognitive impairment.

* Percentages rounded to the nearest integer.

#### Observational studies

Of the twenty-two included studies, thirteen were observational studies, including five cohort, six cross-sectional and two case–control designs (Table 3). Most were conducted in China (*n* 6), including both case–control^(25,66)^ and three of four cross-sectional studies^(29,55,67)^. Observational studies were also the only study type representing Asian populations. While no observational studies evaluated adolescent populations, senior populations were included by all studies of this type, six of which also included adults. Three studies included only female subjects^(68–70)^, with the rest including both males and females. At baseline, most studies included individuals without any cognitive-related impairments; however, case–control studies evaluated individuals with MCI^(25,66)^, and one cross-sectional study reported subjects with a high risk of developing MCI^(24)^. Across all thirteen studies, the sample size of analysed subjects ranged from 87 to 30,484, with a maximum follow-up time of 23 years in the case of cohorts. No studies were found in populations from South America, Africa, or Oceania.

**Table 3:** Study characteristics of observational studies

References	Characteristic			Red meat category			
		Total count		Red meat, includes beef		Red meat, unspecified	
		*n*	%*	*n*	%*	*n*	%*
		13	100	5	38	8	62
Design							
^(23,27,68–70)^	Observational, cohort	5	38	2	40	3	38
^(25,66)^	Observational, case–control	2	15	1	20	1	13
^(24,29,55,67–73,77)^	Observational, cross sectional	6	46	2	40	4	50
Age category†							
^(23,25,55,66,67,77)^	Adult (18–64 years)	6	46	2	40	4	50
^(23–25,27,29,55,66–70,73,77)^	Senior (65+ years)	13	100	5	100	8	100
Cognitive status at baseline							
^(24,25,66)^	MCI or risk of CI	3	23	1	20	2	25
^(23,27,29,55,67–70,73,77)^	No impairments	10	77	4	80	6	75
Participant region							
^(23,25,27,29,55,66,67)^	Asia	7	54	3	60	4	50
^(24,69,73)^	Europe	3	23	1	20	2	25
^(68,70,77)^	North America	3	23	1	20	2	25
Sample size							
^(24,25,29,66,73)^	<500	3	38	2	40	3	38
^(55,67,69)^	500–<5000	3	23	2	40	1	13
^(70,77)^	5000–10 000	2	15	1	20	1	13
^(23,27,68)^	>10 000	3	23	0		3	38
Duration							
^(24,25,29,55,66,67,73,77)^	Cross-sectional or case–control	8	62	3	60	5	63
^(25,68)^	5–<10 years	2	15	1	20	1	13
^(27,69)^	10–<15 years	2	15	1	20	1	13
^(23)^	>20 years	1	8	0		1	13

MCI, mild cognitive impairment; CI, cognitive impairment.

* Percentages rounded to the nearest integer.

† Not exclusive categories.

### Beef and red meat intake

#### Intervention trials

**Fig. 2:**
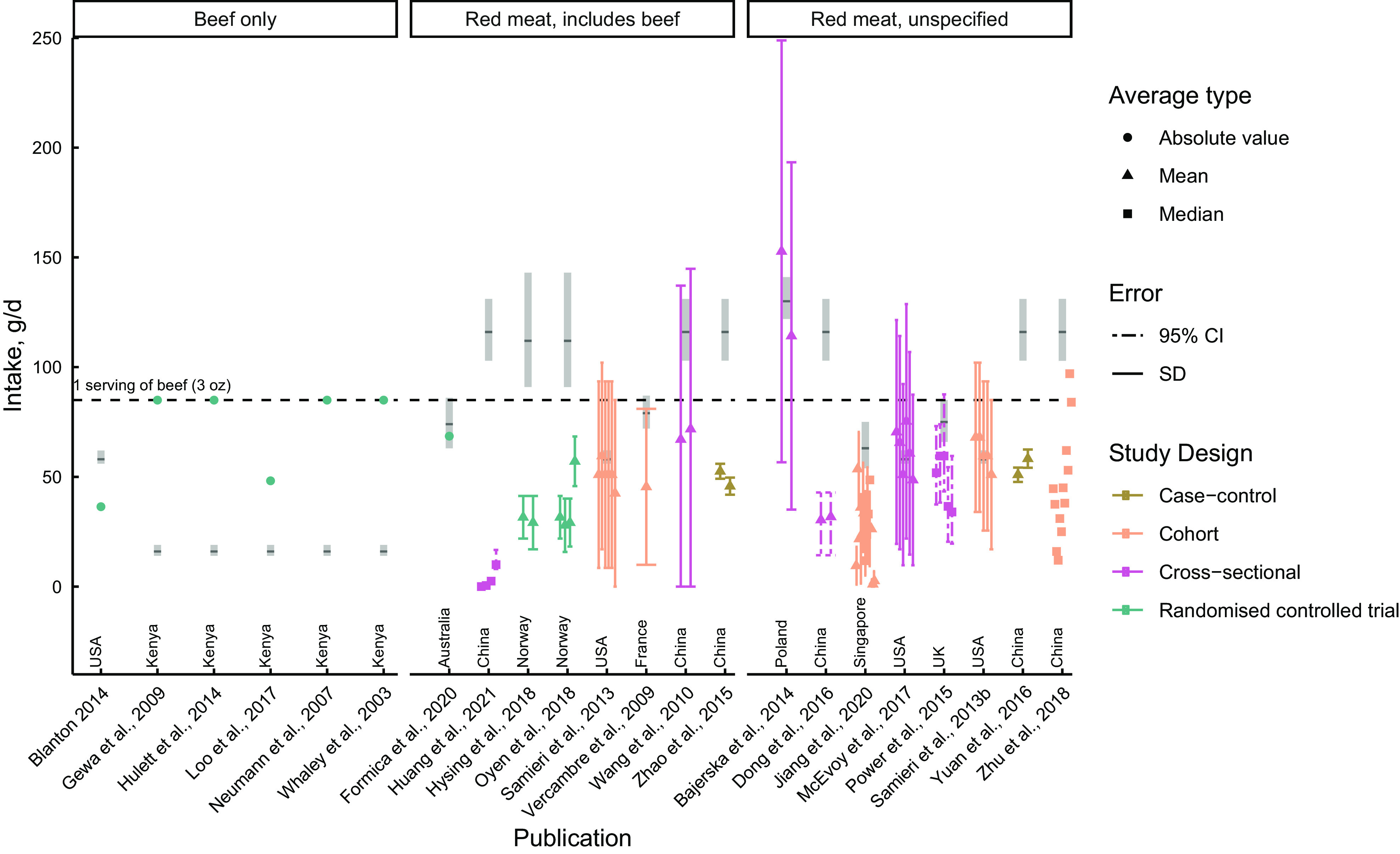
Reported beef or red meat intake (in g/d) across included studies. Reported intake of beef for each point represents the intake of an individual group or quintile reported in each study. The shaded bars represent the mean and 95 % CI for total red meat intake estimated from Miller *et al.* ^(71)^, which includes unprocessed red meat and processed red meat (including poultry).

Most of the RCT specifically evaluated beef intake ranging from 36·42^(58)^ (calculated) to 85 g/d^(59,63–65)^ (Fig. 2). Although six of the nine intervention trials^(54,58,59,63–65)^ evaluated beef only, others focused their evaluations on red meat intake. Of these studies reporting red meat, beef was explicitly stated as part of red meat intake and total intake of red meat here ranged from 27·93^(52)^ to 68·57 g/d^(28)^.

While RCT did quantify the prescribed amount of beef consumed by subjects in the intervention, there was a noted lack of follow-up measurement of beef intake from the total diet throughout the duration of trials. For example, in a study providing subjects biscuit containing dried beef^(54)^, there was no report of total dietary intake in the background diet over the duration of the trial. Blanton and colleagues provided women with 85 g beef three times per week^(58)^ and asked subjects not to consume beef at other meals more than once every other week. Although total dietary intake was validated through a food frequency questionnaire (FFQ), the authors reported nutrient consumption, rather than total beef consumption. The lack of adequately reporting red meat intake from the subjects’ background diet makes the derivation of total beef consumed over the period challenging as the intakes listed in the interventions are likely underestimations of actual beef intake. It remains crucial that researchers both measure and report total dietary intake of the intervention food over the duration of a trial.

When considering total beef consumption in the USA, Agarwal and Fulgoni^(22)^ report intake as 45·6 g per adult per day spanning 2011–2018, a fairly close estimate and 9 g higher than reported by Blanton, the only RCT performed in the USA^(58)^. Despite beef intake from this study not being far from national estimates, it was about 22 g less than the reported total global intake for red meat in the USA, which was estimated as 58 g/d^(71)^. This is similar to the reported intake for processed and unprocessed red meats from a nationally representative sample of the US population, which was 14 lean ounce-equivalents per week, or 56·68 g/d^(72)^. Other RCT performed in Kenya provided participants with amounts of beef far greater than global estimations of total red meat, with participants receiving between 60 and 85 g/d, while the estimated total red meat intake for this region was about 16 g/d^(71)^. It is interesting to note that despite the estimated total red meat intake amount as reported by Miller *et al*.^(71)^ including poultry, it is still vastly lower than amounts consumed by subjects in the CNP trial. This discrepancy between intakes may be explained by the fact that trials frequently administer higher amounts of the exposure to detect a meaningful effect, instead of mimicking regionally representative intakes. Due to lack of available data specifically on beef intake in Kenya, in combination with limited red meat intake data to verify current estimates, the accuracy of these estimations remains unclear.

In contrast with the interventions conducted in Kenya, two RCT conducted in Norway^(52,53)^ provided subjects with far less red meat than estimated average intakes of total red meat for this region^(71)^. Subjects were provided with 50 to 80 g of fatty fish or meat (chicken/lamb/beef) three times per week, calculated as about 21–34 g/d; however, subjects were allowed to consume red meat within their background diet throughout the trial, resulting in a total red meat intake post-treatment of closer to 57·08 g/d in the red meat group. Although this intake amount is closer to regional estimates than are baseline (27·9 g/d) or treatment intakes, it still remains lower than the regional red meat intake, estimated between 91 and 143 g/d. Lastly, only one RCT evaluated beef intake at a regionally representative intake level^(28)^. Subjects were provided with an equivalent of about 68 g/d, which was comparable to the estimated red meat intake for Australians between 63 and 86 g/d.

#### Observational studies

None of the observational studies evaluated beef specifically, rather, they listed beef consumption as part of a larger ‘red meat’ category in the overall diet. Five studies specified that beef was a component of the ‘red meat’ dietary category, while the others only specified ‘red meat’.

Average intake of red meat ranged from 0·5^(67)^ to 152·75 g/d^(24)^; however, most studies reported averages of less than 85 g/d (Fig. 2). Not only were there great variations in average intake of red meat, but the precision of the estimates also differed substantially between studies. For example, Zhao *et al.* ^(66)^ demonstrated CV of 6·5–8·5 % with a total sample size of 404, whereas mean estimates from Bajerska *et al.* ^(24)^ had much higher CV of up to 69 % despite a much smaller total sample size of 87. While study designs and methodologies are heterogenous, such vast differences in precision are still striking.

In comparison with global total red meat intake, four studies^(24,68,70,73)^ closely matched reported total red meat intake or had a range that encompassed the global intake estimates for their respective geographical location. These included red meat intake for the USA, Poland and the UK. However, the global intakes for total red meat per country were more frequently higher than what was reported in observational studies^(25,27,29,55,66,67)^, and interestingly, all six observational studies from China reported far less red meat consumption than estimated red meat intake for this region^(71)^. For example, in one cohort study^(27)^, even the highest quintiles of red meat intake for men and women (97 and 84 g/d) were still 19 and 32 g/d lower than the global estimated average for China, inclusive of the reported 95 % CI (103, 131 g/d)^(71)^. It is possible that the inclusion of poultry in the estimation of global red meat intake contributed to an inaccurate estimation of total red meat intake^(71)^. This consistent difference between reported red meat intake in studies *v*. estimated intake for this region may further be due to the evaluated age category of interest. All studies evaluating Chinese populations evaluated adults and seniors, with two of the studies focusing their evaluation on elderly subjects. Albeit not pertaining to Asian regions, NHANES data have shown that elderly persons consume larger amounts of meat than do adolescents^(74)^. Regardless of the source of this difference, it is possible either the observational data are a more reliable representation of actual red meat intake than estimated total red meat intake for this region, or the responses from the FFQ are inaccurate estimations of true intake. Interestingly, none of the included observational studies reported red meat intake at levels higher than the estimated intake for the country. Again, the inclusion of poultry intake in the global red meat intake estimations^(71)^ may be an explanation for this.

The method of ascertainment of dietary intake across studies was overwhelmingly by means of FFQ. Though dietary assessment tools vary in their objective and data gathered per food group, FFQ tend to lack a degree of granularity in defining food groups and instead aim to capture dietary patterns over time rather than quantitative intake of specific food groups. FFQ additionally require both adaptation for use in different geographical locations and validation before use. Validation is often performed by 24-h dietary recall, as seen in Zhu *et al.* ^(27)^; however in this particular case, the correlation represented only a moderate relationship between the FFQ and 24-h dietary recalls. Research by Steinemann *et al.* ^(75)^ further evaluated the relationship between FFQ and shorter-term dietary recall, such as 4-d dietary recall, and showed that out of twenty-five food groups, meat was one of the food groups with the highest recall (in both FFQ and 4-d recall), second only to alcohol. Although this finding shows that subjects generally have an acceptable recall of meat intake and subsequently may support the use of FFQ, it simultaneously demonstrates that FFQ may be more appropriate for measuring major food groups, such as ‘meat’ or ‘fruits’ and may not be precise enough to measure the consumption of specific sources or types of foods. Results from this review highlight their finding, as can be seen in the limited specificity in the current reporting of beef and red meat intake forms, as well as the observed heterogeneity in how beef and red meat forms are reported. The study by Steinemann *et al.* ^(75)^ also revealed that subjects reporting their consumption of meat through FFQ over-estimated their consumption by about 30 % compared with 4-d recall. It is possible that red meat intake reported by studies included in this review are similarly over-estimates of actual intake^(73)^.

There is a further considerable limitation in the use of FFQ when subjects are required to recall dietary intake from memory over long periods of time^(76)^ particularly when studies include participants with MCI. Despite FFQ being the most common tool to measure dietary intake in large cohort studies^(75)^, the increased application of validation of FFQ through 24-h recalls or weighed food records, particularly in studies evaluating dietary intake and cognition-related outcomes, could allow for the collection of more accurate data, while simultaneously reducing the incidence of potential recall bias.

### Cognitive outcomes

**Fig. 3:**
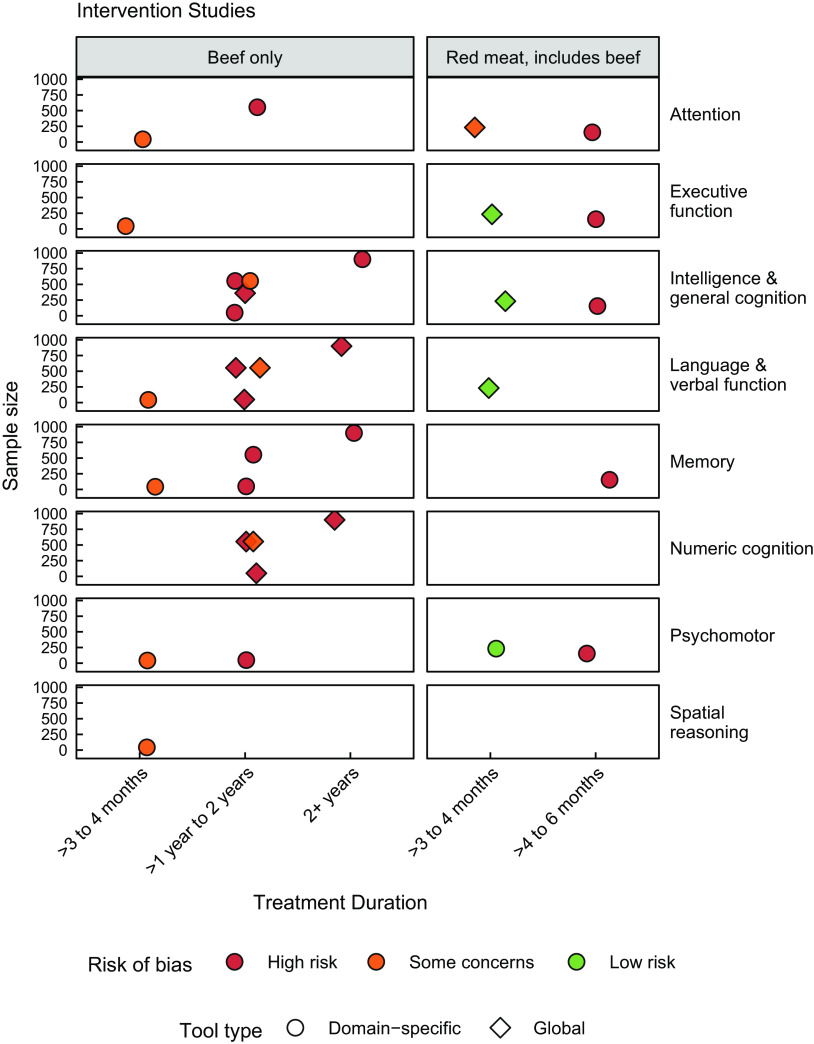
Plot of studies by intervention duration and sample size, per cognitive domain. Each data point represents a single study. Circles represent studies evaluating outcomes using domain-specific tests, and diamonds represent studies evaluating outcomes using global tests

**Fig. 4:**
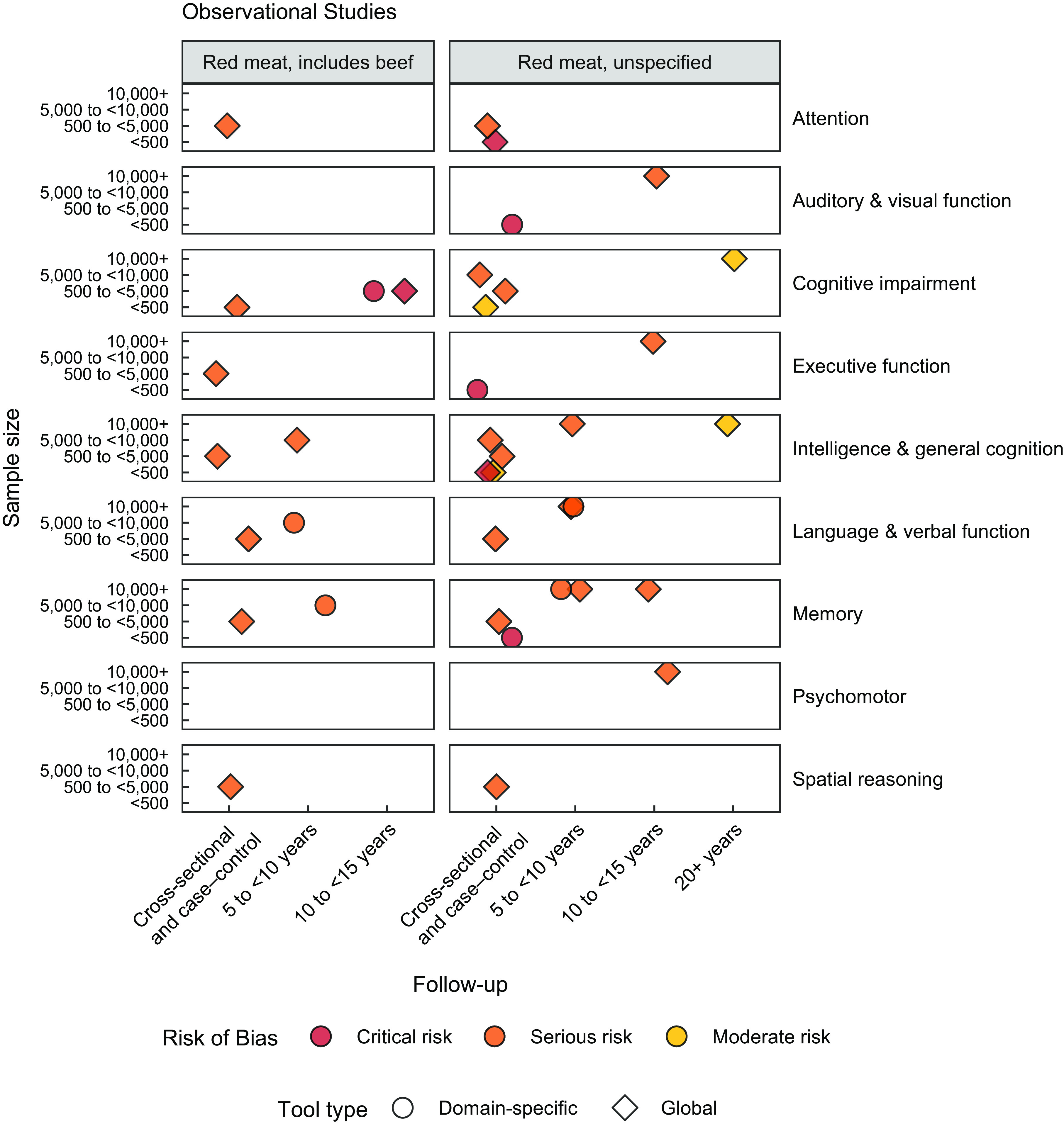
Plot of studies by follow-up time and sample size, per cognitive domain. Each data point represents a single study. Circles represent studies evaluating outcomes using domain-specific tests, and diamonds represent studies evaluating outcomes using global tests

Cognition-related outcomes as reported by the authors were binned into one or multiple of the following pre-specified cognitive domains: memory, executive function, language and verbal function, attention, psychomotor, spatial reasoning, intelligence and general cognition, numeric cognition, and auditory and visual function (Figs. 3 and 4). Although the review team did assess the specific domains and sub-domains that each cognitive assessment tool measures, what is shown here is rather cognitive domains as reported in the results sections of studies as a means to further highlight the discrepancy in current reporting between authors utilising the same tool(s). For example, authors implementing the MMSE tool mostly reported total scores which we categorised under intelligence and general cognition^(23,24,68,70,73,77)^; however, some authors additionally reported outcomes categorised under language and verbal function and memory^(68)^ or as a diagnostic of cognitive impairment^(23,73,77)^. To further illustrate this variation in reporting, some reports using the Montreal Cognitive Assessment (MoCA) reported measures spread across attention, memory, intelligence and general cognition, spatial reasoning, and language and verbal function^(55,67)^; however, one of these studies additionally evaluated executive function^(67)^, while the other did not^(55)^. In other words, while the same tool has been used across numerous studies, the same cognitive domains should have been measured and reported across all studies, but this was not the case.

While the above demonstrates the variety in cognitive domain-related reporting between studies using the same cognitive tools, there was also a discrepancy within studies and how cognitive test results were reported. Blanton *et al.* ^(58)^ is exemplary in that authors report both the methods and results for each task assessed by the use of the Cambridge Neuropsychological Test Automated Battery (CANTAB) to measure domains such as memory, executive function, spatial reasoning and attention. Conversely, Samieri *et al.* ^(68)^ reports using the digit span backwards test to measure attention; however, results of performance on the digit span backwards test were pooled with performance on other tasks under a ‘global cognitive score’, and thus any nutritional impact on attention specifically cannot be derived. On the other hand, rather than assessing cognition as an end point in itself, seven studies used screenings tools such as the MoCA or MMSE to assess cognitive impairment^(23,25,29,55,66,73,77)^ and then assessed red meat intake between subjects with varying levels of cognitive impairment. It is evident that the depth and breadth of cognitive assessments vary significantly in the literature and interpretations of the data in Figs. 3 and 4 must thereby be performed with consideration for the fact that the different domains do not signify the variation in the cognition assessment tools used, but rather the variation in the reporting of the tools.

As an additional means to identify gaps in the evidence, reported cognitive assessment tools were assigned as either global or domain-specific in nature (see online Supplemental Table 4). Global assessments typically determine a wide range of cognitive abilities as a means of assessing everyday function, without in-depth evaluation into specific functions. Such tools will normally require many underlying cognitive functions like attention, working memory, executive function, motor skills and more but are not designed for deep assessments of those sub-domains. In contrast, domain-specific assessment tools, such as CogState, Raven’s Progressive Matrices and Beery Test of Visual-Motor Integration, are designed to test an individual sub-domain of or tasks related to cognition, such as those performed by Loo *et al.* ^(54)^ and Formica *et al.* ^(28)^. Results from this review showed that studies specifically measuring beef intake tended to use domain-specific tools (Fig. 3), studies measuring red meat intake where beef was specified as part of red meat used both domain-specific as well as global assessment tools, with a slight tendency towards global tools (Figs. 3 and 4). Lastly, studies that only reported red meat intake without further specification almost solely measured cognitive outcomes through global tools (Fig. 4). Overall, studies that evaluated red meat intake did not use tools designed to assess specific cognitive domains.

Collectively, the most common assessment tool reported among the twenty-two included studies was the MoCA, followed closely by the MMSE or a variation thereof (cognitive tool per study available in online database at https://github.com/Traverse-Science/Beef-and-Cognition-SR). The most frequently measured cognitive outcomes were intelligence and general cognition, memory, and language and verbal function. Auditory and visual function was reported only by studies evaluating red meat without specification of beef inclusion (Fig. 4), and interestingly, red meat without specification of beef inclusion was the only intake category where numeric cognition was not assessed. Studies assessing beef intake tended to look at cognitive domains such as executive function, language, numeric cognition, and memory, and focused less on other components of cognition such as attention and spatial reasoning (Fig. 3). Provided the CNP trials performed their evaluations on school-aged children and formed a large portion of the beef intake data, results appear to be clustered around these cognitive domains relevant to school-aged children for intervention trials, that is, intelligence and general cognition, as well as numeric cognition and verbal function (Fig. 3). The remaining trials included the evaluation of other domains such as executive function, spatial reasoning and psychomotor function.

### Risk of bias

#### Intervention trials

**Fig. 5:**
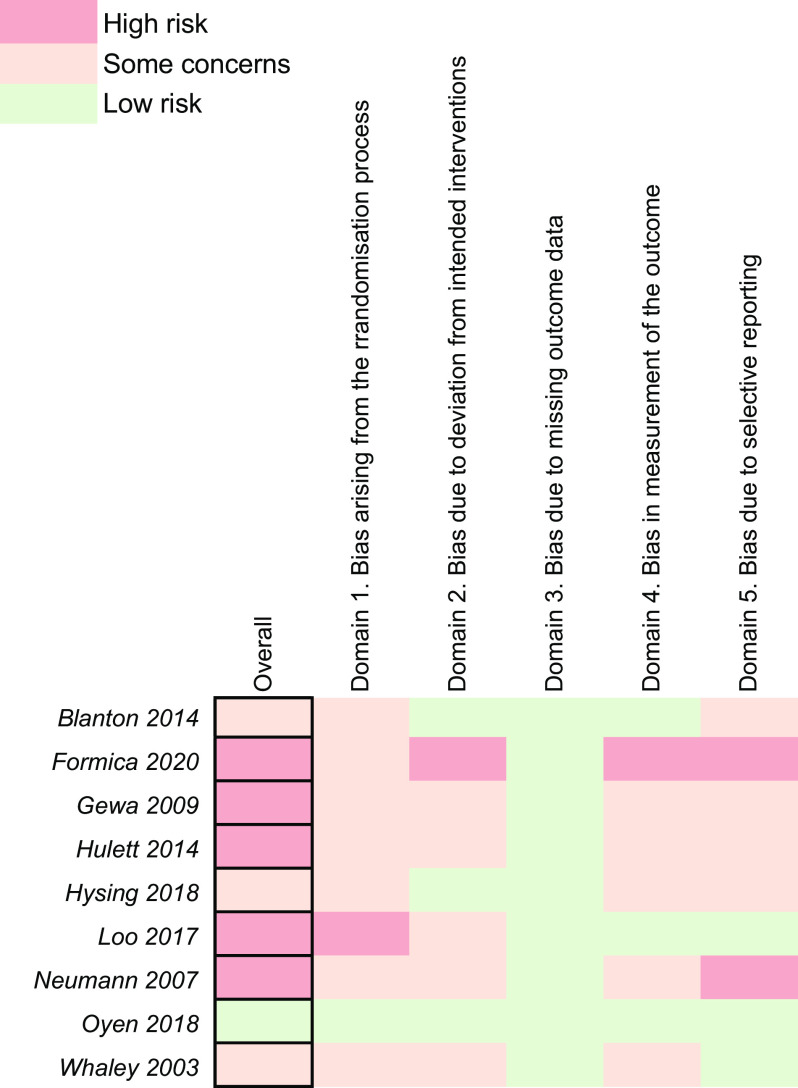
Risk of bias assessment for intervention trials

Of the nine RCT, five studies were rated with high risk of bias^(28,54,59,64,65)^, three were rated with some concerns^(53,58,63)^ and one was rated with low risk of bias^(52)^ (Fig. 5). Overall, intervention trials rated well within domain 3 (bias due to missing outcome data), owing to the relative completeness of the data and in cases of missing data, this was adequately addressed in the analysis. In contrast, there is much room for improvement within domains 1, 2, 4 and 5, which contributed negatively to overall ratings. Particularly domain 1 (bias arising from the randomisation process), which entails bias assessment due to the randomisation process. Groups were seldom concealed to treatment allocation, and on occasion unbalanced in size^(54,64)^. This was further evident through significant differences in baseline characteristics^(28)^; however, these factors were adjusted in the analyses. The largely negative contribution of domain 2 (bias due to deviation from intended interventions) to the overall rating of interventions was due to participants and persons delivering interventions not being blinded to the intervention.

One study was able to blind participants to the intervention^(54)^ by converting beef to a powdered form and then baking it into biscuits. While this approach adequately blinds subjects and researchers, it entails greater processing which may impact the bioactive components in beef that are critical to the outcomes evaluated. Ultimately increased risk due to blinding procedures is to be expected. Blinding is a significant issue with the trials reported here and in nutrition trials on whole foods in general, and one that must be considered in the interpretation of results.

Lastly, many RCT did not provide proof of a pre-specified analysis plan, which is a recognised measure of bias in the RoB2.0 tool. As discussed above, some tools that measure multiple cognitive domains were used; however, only a selection of the results were reported in either the main text or supplemental material.

#### Observational studies

**Fig. 6:**
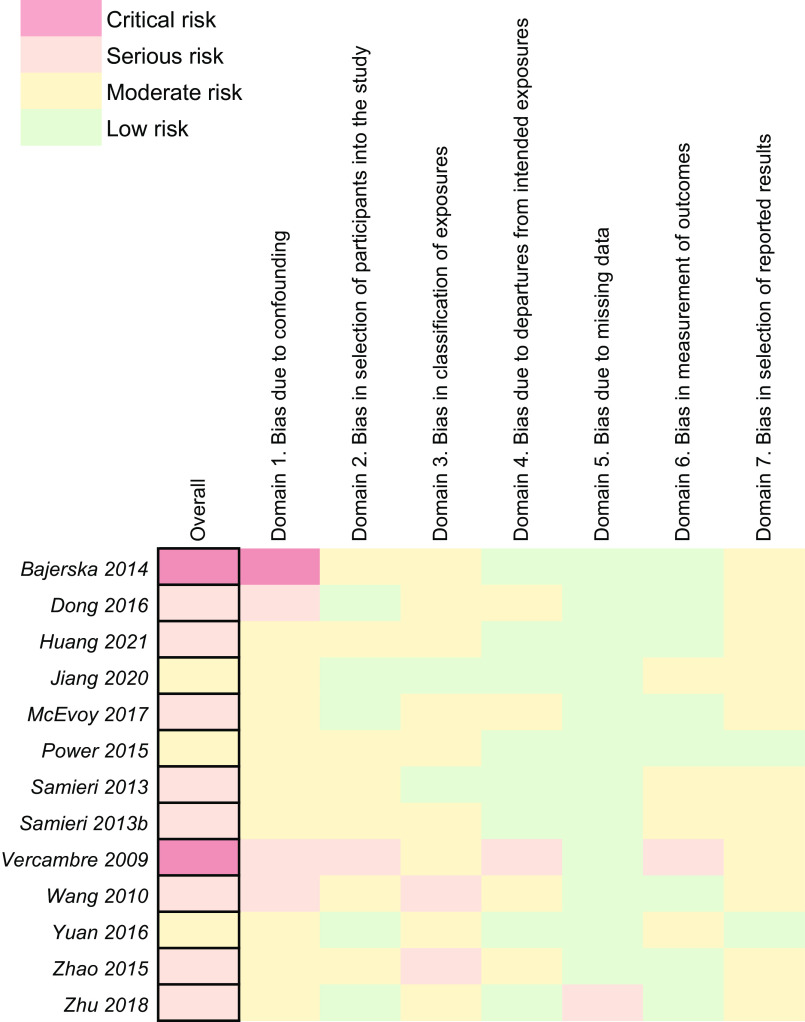
Risk of bias assessment for observational studies

Of the thirteen observational studies, two studies were rated as critical risk^(24,69)^, eight as serious risk^(27,29,55,66–68,70,77)^ and the remainder with moderate risk^(23,25,73)^ (Fig. 6). Bias due to confounding was present in all studies, and although this can be ascribed to inappropriate adjustment for confounding factors in many cases, the RoBNObs tool has been constructed in a manner by which a study can only receive a low-risk rating if no confounding is expected. This was unobtainable by the study designs included in this review. As confounding can always be expected in nutritional epidemiology, consideration should instead be placed on the extent of adjusting and controlling for those variables. One study^(24)^ obtained a critical risk rating for this domain due to participants being elderly, located in rural areas, with high risk of metabolic syndrome, and recruited from one general practitioner. While these factors do not make it a poor study on its own, the study does not generalise well to other populations and controlling for confounds was not possible.

Overall, there was no concern for bias due to departures from intended exposures, largely due to the exposure of interest being general dietary intake, and it was seldom reported that subjects deviated from their routine daily intake. Similarly, bias due to missing data was not a domain of concern owing to the cohorts comprising a large number of participants, where missing data was never more than 5 % of the total cohort size. For domain 7 (bias in selection of reported results), the higher risk ratings largely stemmed from observational trials not pre-registering protocols. Future observational trials on the subject could achieve lower risk ratings by making the following low-cost adjustments: greater statistical adjustment of confounds, clearer reporting of the exposure (by specifying the type of beef and greater detail on cuts, fat %, grade, etc.) and pre-registration of study protocols.

### Limitations

This scoping review was limited by the inability to assess scope-fit by titles and abstracts alone, as the requirement that beef intake be quantified typically required full-text screening. For this reason, backwards citation screening was implemented to supplement the use of PubMed. Similarly, while there are numerous reports of the relationship between beef intake and cognition, several of these studies either did not quantify or adequately describe beef intake. For these reasons, half of the full texts screened were excluded. That said, secondary analyses of publicly available data may alleviate limitations in the present review and address concerns that arose from the risk of bias assessment. Further noted limitations of this review were the use of a single database for the search of relevant articles. Excluding studies that assessed beef intake and cognition, without quantifying the intake of beef in a calculable manner, could be considered both a strength and a limitation of this review.

## Conclusions

The dearth of evidence relating red meat and beef to cognition, further complicated by the lack of clearly defined and differentiated red meat and beef groups, makes the assessment of the impact of these intake groups on cognition complex. This explains in part the diversity in results reported in reviews and meta-analyses on the subject. As the study of beef intake and cognition is young and relatively understudied, future studies can make significant progress in the field primarily by improving the standards of reporting study methodology, describing the exposure in greater detail and further adjusting for confounds. Despite a greater body of evidence relating red meat intake and cognition, future studies will benefit from the same guidelines as above. While the variety of geographical populations assessed is a strength of this body of evidence, greater research is warranted in child and adolescent populations for both red meat and beef intake. Greater RCT are required, and these should strive to replicate realistic consumption patterns in the population of interest. The publicly available database created here will allow for independent interpretation of results to draw conclusions relevant to specific questions regarding red meat and beef consumption and cognitive outcomes.

## Supplementary material

For supplementary material accompanying this paper visit https://doi.org/10.1017/S1368980023001933
